# Comparing the Efficacy and Safety of Induction Therapies for the Treatment of Patients with Proliferative Lupus Nephritis in South Africa

**DOI:** 10.1155/2020/2412396

**Published:** 2020-10-19

**Authors:** Phelisa Sogayise, Udeme Ekrikpo, Ayanda Gcelu, Bianca Davidson, Nicola Wearne, Ugochi Okpechi-Samuel, Theophilus Ifeanyichukwu Umeizudike, Innocent Ijezie Chukwuonye, Ikechi Okpechi

**Affiliations:** ^1^Department of Medicine, University of Cape Town, Cape Town, South Africa; ^2^Department of Nephrology, University of Uyo, Uyo, Nigeria; ^3^Kidney and Hypertension Research Unit, University of Cape Town, Cape Town, South Africa; ^4^Division of Rheumatology, Department of Medicine, University of Cape Town, Cape Town, South Africa; ^5^Division of Nephrology and Hypertension, University of Cape Town, Cape Town, South Africa; ^6^Department of Internal Medicine, Federal Medical Centre, Jabi, Abuja, Nigeria; ^7^Nephrology Unit, Department of Medicine, Lagos State University Teaching Hospital, Ikeja, Lagos, Nigeria; ^8^Department of Medicine, Federal Medical Centre, Umuahia, Nigeria

## Abstract

**Background:**

Lupus nephritis (LN) can be complicated with requirement for kidney replacement therapy and death. Efficacy of induction therapies using mycophenolate mofetil (MMF) or intravenous cyclophosphamide (IVCYC) has been reported from studies, but there is limited data in Africans comparing both treatments in patients with proliferative LN.

**Methods:**

This was a retrospective study of patients with biopsy-proven proliferative LN diagnosed and treated with either MMF or IVCYC in a single centre in Cape Town, South Africa, over a 5-year period. The primary outcome was attaining complete remission after completion of induction therapy.

**Results:**

Of the 84 patients included, mean age was 29.6 ± 10.4 years and there was a female preponderance (88.1%). At baseline, there were significant differences in estimated glomerular filtration rate (eGFR) and presence of glomerular crescents between both groups (*p* ≤ 0.05). After completion of induction therapy, there was no significant difference in remission status (76.0% versus 87.5%; *p*=0.33) or relapse status (8.1% versus 10.3%; *p*=0.22) for the IVCYC and MMF groups, respectively. Mortality rate for the IVCYC group was 5.5 per 10,000 person-days of follow-up compared to 1.5 per 10,000 person-days of follow-up for the MMF group (*p*=0.11), and there was no significant difference in infection-related adverse events between both groups. Estimated GFR at baseline was the only predictor of death (OR: 1.0 [0.9–1.0]; *p*=0.001).

**Conclusion:**

This study shows similar outcomes following induction treatment with MMF or IVCYC in patients with biopsy-proven proliferative LN in South Africa. However, a prospective and randomized study is needed to adequately assess these outcomes.

## 1. Introduction

Systemic lupus erythematosus (SLE) is a chronic multisystem autoimmune disorder with a predilection for young females. Lupus nephritis (LN) is a significant cause of morbidity and mortality in SLE patients since up to 60% of adults with SLE develop LN [1]. Several studies have shown a clear and strong association between developing LN and mortality and other poor patient outcomes [2–4]. One large study from South Africa reported that presence of LN was the only factor associated with mortality in 226 SLE patients, with a 5-year survival rate of 60% [2]. The goal of the management of LN is to achieve the best possible clinical outcome with complete disease remission and minimal toxicities using appropriate immunotherapies which can be associated with adverse effects, especially life-threatening infections [5]. Although the use of immunotherapies continue to improve the prognosis of SLE patients, a significant proportion still progresses to end-stage kidney disease (ESKD) within a decade of diagnosis of LN [6, 7]. There is evidence that people of African ethnicity are at greater risk of a severe form of lupus and a higher risk of progression to kidney failure [8, 9].

In the landmark multinational Aspreva Lupus Management Study (ALMS), there was no significant difference between both groups regarding response to treatment and adverse events [10]. However, in a group that was classified as “other” that included people of African ancestry, patients treated with MMF had a significantly higher rate of response than those treated with IVCYC (*p*=0.033) [10]. Several studies have since compared efficacy and outcomes between both therapies in different populations with mixed findings [11–14]. Although some studies from South Africa [15–18] have descriptively reported on various treatment outcomes in patients with confirmed LN, there are no studies from the region that have reported efficacy or survival outcomes based on comparison of treatment between both therapies. Our study aim was to assess proportion of patients achieving complete remission based on induction therapies with either MMF or IVCYC from a single centre in South Africa.

## 2. Methods

### 2.1. Study Population

This study protocol was approved by the joint Human Research Ethics Committee of the University of Cape Town. The study had a retrospective design and was conducted at the Division of Nephrology and Hypertension, Groote Schuur Hospital (GSH), Cape Town, South Africa. The study included all patients with biopsy-proven proliferative LN and who received induction treatment with either IVCYC or MMF over a 5-yrear period at the Division of Nephrology and Hypertension, University of Cape Town. Patients included in the study were those with consecutive biopsy-confirmed proliferative lupus nephritis (classes III, IV, and V mixed type) classified according to the ISN/RPS criteria [19].

### 2.2. Data Collection

We collected relevant biodata at baseline, 6 months, 12 months, and at last follow-up visit on patient demographics, clinical features (e.g., blood pressure, use of ACE-inhibitors, and induction with IVCYC or MMF), biochemical parameters (serum creatinine, estimated glomerular filtration rate, urine protein-creatinine ratio, and autoimmune biomarkers), and baseline histological features. The estimated glomerular filtration rate (eGFR) was calculated using the abbreviated Modification of Diets in Renal Disease (MDRD) [20]. Information from biopsy reports including number of glomeruli, presence of interstitial fibrosis, number of crescents if present, and histology class as reported by the pathologist was extracted. Adverse effects of treatment were recorded based on overseeing physician documentation of relatedness of such features with treatment given to patients. Hence, these were limited to the occurrence of infections, tuberculosis, diabetes (steroid-related), and death in both groups.

### 2.3. Variable Measurement

Treatment response was measured by assessment of proteinuria and serum creatinine at regular intervals. Response to therapy in LN was assessed as complete remission (CR), partial remission (PR), or no remission. These outcomes were defined based on the Kidney Disease Improving Global Outcomes (KDIGO) guidelines as follows [21]:

- Complete remission (CR): return of serum creatinine to the previous baseline plus a decline in the urinary protein to creatinine ratio (uPCR) to <0.5 g/24 hours.
- Partial remission (PR): stabilisation (25%) or improvement of serum creatinine, but not to normal, plus *a* > 50% decrease in uPCR. If there was nephrotic range proteinuria (uPCR > 0.3 g/24 hours), the improvement required a 50% reduction in the uPCR.
- Deterioration was defined as a sustained 25% increase in the serum creatinine.
- Relapse was defined as clinical manifestations indicating activity, namely, active urinary sediments, increasing proteinuria with or without serological reactivation in a patient who was previously in complete or partial remission.

### 2.4. Statistical Analysis

Statistical analysis was performed using Stata 15.1 (StataCorp, TX, USA). The Student's *t*-test was used to compare means between the two groups if normally distributed, while the Mann–Whitney *U* test was used for comparison in cases of deviation from normality. The Chi-square was used for the statistical comparison of proportions between two groups. Univariable and multivariable logistic regression models were used to identify independent associations with remission. Kaplan–Meier graphs were used to show the time-to-mortality experience of different groups within the cohort with the log-rank test used to compare survival experiences statistically. Cox proportional hazard models were used to identify independent predictors of mortality in the cohort. A significant *p* value was taken as *p* < 0.05.

## 3. Results

### 3.1. Baseline Features

Eighty-four patients with proliferative LN were included in the analysis. Overall, the mean age was 29.6 ± 10.4 years with a female preponderance (88.1%). There was also a preponderance of patients of mixed ancestry (67.8%) but there was no significant difference in racial distribution of patients (*p*=0.86). Although mean systolic and diastolic blood pressures were higher in those treated with IVCYC than in those treated with MMF, only the mean arterial pressure was noted to be significantly higher in the IVCYC group (*p*=0.04) (Table 1). Other baseline features (for the groups and overall) are shown in Table 1. Overall, the median eGFR was noted to be 69.7 (IQR: 33.5–99.2 ml/min/1.73 m); this was significantly lower in the IVCYC group (*p*=0.02) (Table 1). The median dose of MMF received for those who received this treatment was 1.5 g (IQR: 1.0 g–2.0 g) and those who received IVCYC were treated with 1.0 g–1.5 g/m^2^ body surface area monthly for 6 months, while all patients received 1 mg/kg of oral prednisone (maximum of 60 mg/day) which was tapered monthly to 10 mg/day at 6 months in line with the National Institute of Health protocol which we utilize at our centre.

### 3.2. Histological Features and Complications of Treatment

Overall, there was a median of 14 (10–18) glomeruli per biopsy taken. Overall, there were 42.9% with class III, 41.7% with class IV, and 15.5% with mixed classes, and there was no significant difference in the distribution of the classes between both groups (Table 2). The IVCYC group had a significantly higher proportion of crescents than the MMF group (3.8 [0.0–32.4] versus 0.0 [0.0–6.2]; *p*=0.03). Presence of sclerosed glomeruli or any degree of interstitial fibrosis was not significantly different between the groups. Although infections (upper respiratory tract and urinary tract infections) occurred more frequently in the IVCYC group, there was no significant difference for infections between both treatment groups (*p*=0.20) (Table 2). Other complications that were reported including steroid induced diabetes and tuberculosis were also more frequent in the IVCYC group but were not significantly higher than in the MMF group.

### 3.3. Remission and Relapse

**Figure 1:**
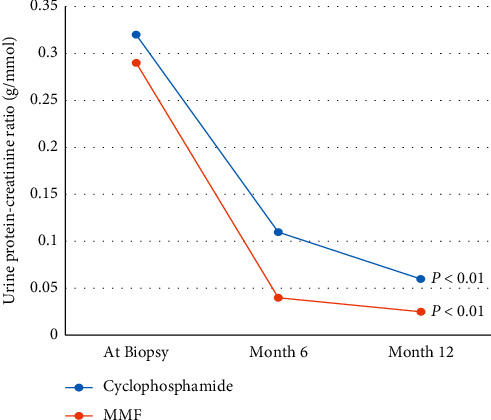
Trend in proteinuria in both groups over the study period (MMF: mycophenolate mofetil).

At six months follow-up, overall, remission (either complete or partial) occurred in 52 (78.8%, 95% CI: 67.0–87.9%) patients with no significant difference between both treatment groups, *p*=0.33 (Table 3). The relapse rate at 12 months of therapy was also not significantly different between both groups; *p*=0.22 (Table 3). No independent predictor of remission emerged to be statistically significant from multivariable analysis (Table 4).  Figure 1 summarizes the trend in proteinuria and shows that both modalities of treatment were significantly associated with reduction of proteinuria.

### 3.4. Mortality

**Figure 2:**
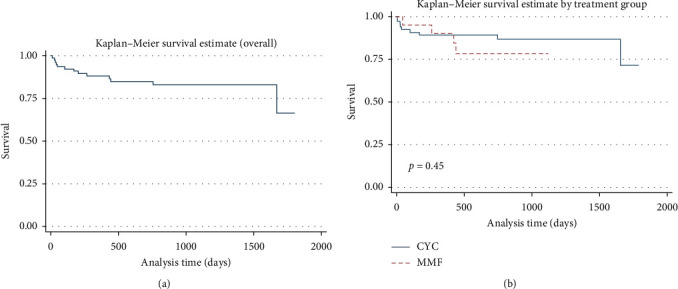
(a) Overall survival and (b) survival comparison between the IVCYC and MMF groups.

A total of 15 deaths were reported; most (14/15) were in the IVCYC group (Table 2). The median time to death was 105 days (IQR 45–267 days) from the date of biopsy. During a total of 32,159 person-days of follow-up, the mortality rate for the IVCYC group was 5.5 per 10,000 person-days of follow-up compared to 1.5 per 10,000 person-days of follow-up for the MMF group (*p*=0.11) (Figures 2(a) and 2(b)). Multivariable analysis showed that eGFR at baseline was the only independent predictor of mortality in this group of patients (Table 4). For every 1 ml/min/1.73 m^2^ increase in eGFR at baseline, there was a 5% reduced risk of mortality in this cohort. Sensitivity analysis, matching both groups by age, gender, ethnicity, and histological class did not reveal any of the variables that predicted outcomes (Table S1).

## 4. Discussion

One of the unmet needs in the treatment of patients with LN in Africa is using cheap, effective, and readily available immunotherapeutic agents with minimal adverse effects. Combination IVCYC and high dose steroids have remained the cornerstone of treatment of LN in Africans leading to various side effects including infections, metabolic derangements, and death [18, 22, 23]. In this study, we report a retrospective comparison of MMF and IVCYC in patients with LN treated in a single Centre in Cape Town, South Africa. The important findings from our study include the following: (i) after 6 months of induction therapy with either IVCYC or MMF, there was no difference in the proportion of patients attaining complete or partial remission between both groups, (ii) there was no significant difference in reported infections-related adverse events between both groups at the last follow-up visit, and (iii) there was no significant difference in mortality between the two treatment groups (*p*=0.11).

In the ALMS study, 370 patients with classes III through V LN were randomized to open-label MMF (target dosage 3 g/d) or IVCYC (0.5 to 1.0 g/m^2^ in monthly pulses) in a 24 wk induction study [10]. Both groups received prednisone, tapered from a maximum starting dosage of 60 mg/d. The primary endpoint was a prespecified decrease in UPCR and stabilization or improvement in serum creatinine. Secondary endpoints included complete renal remission, systemic disease activity and damage, and safety. Overall, there was no significantly different response rate between the two groups (MMF-56.2% versus IVCYC-53.0%; *p*=0.58) [10]. Secondary endpoints were also reported to be similar between both treatment groups; however, further analysis of the trial results showed that in patients with “other” as racial group (mainly Hispanics and African Americans), there was a significantly higher proportion of patients who responded to MMF (60.3% versus 38.5%; *p*=0.033) [10]. In our study, “other” would be mainly Black South Africans and those ethnically classified Coloureds (mixed ancestry) who make up the predominant population in Cape Town. Ethnicity did not play a role in outcome in our study (Table 4).

Several studies comparing MMF and IVCYC for induction in patients with LN have since been published showing varied results but mainly that there is no difference in response rate between MMF and IVCYC treatments for induction [11, 12, 14]. A study from India randomized (equally) 100 patients to IVCYC or MMF for a 24-week induction treatment of LN [14]. Baseline characteristics were similar between both groups; however, proteinuria was significantly higher in the in the IVCYC group and, at 24 weeks, the complete remission rate was 50% in the IVCYC and 54% in MMF group (*p*=0.91) [14]. Our study, conducted in a predominantly African population (black Africans and Africans with mixed ancestry) did not find any difference in response as the complete and partial remission rates were similar between the IVCYC and MMF groups. However, we are aware that this might be a sample size effect given a smaller sample size for the MMF group.

The reported complications associated with treatment were infections (mainly upper respiratory and urinary tract types), pulmonary tuberculosis, and steroid induced diabetes. Although our study did not find any significant difference in complications rate between both treatment groups, most occurred in the IVCYC group (Table 2). This could be an indication of the severity of disease in this group (evidenced by lower eGFR at treatment initiation) or toxicity related to this treatment. For similar reasons, we also found higher mortality in the IVCYC group. In a recent systematic review on complications associated with LN treatment, Thong and Chan [24] included 56 studies (32 randomized controlled trials) and found that MMF as induction treatment was associated with lower overall infection risks than cyclophosphamide in non-Asians (risk ratio 0.60, 95% confidence interval 0.48–0.75, *p* < 0.001). Thus, although infection remains a serious complication during treatment of LN, they concluded that the reported rates and outcomes vary markedly.

Our study has a number of limitations. First, this was a retrospective analysis of those treated in a single centre in South Africa and therefore has all the biases associated with retrospective reviews including inadequate sampling and inability to adequately assess and document all adverse events as they occurred. Second, compliance to treatment was only certain in those who were receiving IVCYC as they had to come in to the clinic to receive the monthly pulse intravenous treatment. Compliance could, however, not be ascertained in the MMF group. Thus, what role this might have played regarding our results is not known. Also, given that lupus disease activity indices are not routinely checked in the Nephrology clinic, we were unable to obtain these scores which we could have correlated with response and relapse rates between both groups. Also, the recent availability of MMF for treatment in the public sector in South Africa may not have allowed sufficient time for us to fully assess its efficacy and safety in our patient population. Finally, the choice of induction therapy was solely based on physician preference given that those with more severe presentation, evidenced by significantly lower eGFR, were more likely to have been treated with IVCYC. These differences were, however, accounted for in multivariate analysis to determine predictors of remission and death (Table 4). Since its discovery, IVCYC has been the mainstay of therapy for LN (especially in Africa) given that it is readily available and cheap. The low utilization of MMF in our study is related to high cost. However, with availability of generic formulations and ability to monitor MMF levels in blood, it is expected that use of MMF will likely increase for the treatment of LN. Despite these limitations, our study is the first in South Africa where the prevalence of LN is highest amongst all sub-Saharan African countries. Therefore, our results provide evidence to clinicians for use of either MMF or IVCYC in treating patients with proliferative LN. We still recommend adequate monitoring of patients for infections and other side effects related to these therapies.

## 5. Conclusion

In South Africans with proliferative LN, our study has found no difference in response rate, relapse rate, or occurrence of adverse effects to induction therapy with MMF or IVCYC. However, prospective randomized studies to test our findings are still needed.

## Figures and Tables

**Table 1:** Baseline demographic and clinical characteristics of study participants.

	IVCYC (*n* = 65)	MMF (*n* = 19)	Total (*N* = 84)	*p* value
Mean age ± SD (years)	30.3 ± 11.2	27.0 ± 6.9	29.6 ± 10.4	0.23
Female sex (%)	74.3	25.7	88.1	0.07
Ethnicity (%)				
** **Black	30.7	31.5	30.9	0.08
** **Mixed ancestry	67.6	68.4	67.8	
** **White	1.5	0.0	1.1	
Mean SBP (at biopsy) (mmHg)	131.7 ± 20.5	122.9 ± 13.6	129.7 ± 19.4	0.08
Mean DBP at biopsy (mmHg)	80.1 (14.2)	73.2 ± 10.7	78.5 ± 13.7	0.05
mABP (at biopsy) (mmHg)	97.4 ± 15.0	89.7 ± 10.9	95.7 ± 14.5	**0.04**
Use of ACE-I/ARB (%)	89.2	94.7	90.5	0.47
Hb at biopsy (g/dl)	9.9 ± 2.8	10.6 ± 2.0	10.1 ± 2.6	0.36
WBC at biopsy ( x 10^9^/L)	8.1 ± 4.5	6.7 ± 3.5	7.8 ± 4.3	0.23
Low C3 (%)	87.5	78.9	85.5	0.35
Low C4 (%)	69.8	79.0	72.0	0.44
Positive ANA (%)	86.9	88.9	87.3	0.82
Positive dsDNA (%)	90.3	84.2	88.9	0.46
Serum albumin (g/L)	28.2 ± 6.9	29.5 ± 9.59	28.5 ± 7.5	0.51
Median SCr (IQR) (*µ*mol/L)	96 (65–192)	70 (58–84)	84.5 (62–174)	**0.02**
Median eGFR (IQR) (ml/min/1.73 m^2^)	61.91 (27.9–94.5)	87.2 (69.6–106.8)	69.7 (33.5–99.2)	**0.02**
uPCR (mg/mmol)	320 (185–585)	290 (170–670)	310 (170–590)	0.92

IVCYC = intravenous cyclophosphamide; MMF = mycophenolate mofetil; SBP = systolic blood pressure; DBP = diastolic blood pressure; mABP = mean arterial blood pressure; ACE-I = angiotensin converting enzyme inhibitor ARB = angiotensin receptor blocker; Hb = haemoglobin; WBC = white blood cell count; ANA = antinuclear antibodies; dsDNA = double stranded DNA; SCr = serum creatinine; eGFR = estimated glomerular filtration rate; uPCR = urine protein-creatinine ratio, SD: standard deviation.

**Table 2:** Histopathological data and complications associated with both treatment groups.

	IVCYC (*n* = 65)	MMF (*n* = 19)	Total (*n* = 84)	*p* value
Number of glomeruli (median) (IQR)	13 (9–16)	18 (12–26)	14 (10–18)	0.01
Crescents % (IQR)	3.8 (0.0–32.4)	0.0 (0.0–10.0)	0.0 (0.0–28.6)	**0.03**
Sclerosed glomeruli % (IQR)	0.0 (0.0–5.5)	0.0 (0.0–10.0)	0.0 (0.0–5.5)	0.59
Interstitial fibrosis *n* (%)	32 (50.0)	7 (36.8)	39 (47.0)	0.31
ISN class				
** **III	28 (43.1)	8 (42.1)	36 (42.9)	0.49
** **IV	25 (38.5)	10 (52.6)	35 (41.7)	
** **III + V	2 (3.1)	0	2 (2.4)	
** **IV + V	10 (15.4)	1 (5.3)	11 (13.1)	
IgG deposits				
** **0	15 (23.8)	4 (21.1)	19 (23.2)	0.80
** **1	12 (19.1)	2 (10.5)	14 (17.1)	
** **2	23 (36.5)	8 (42.1)	31 (37.8)	
** **3	13 (20.6)	5 (26.3)	18 (21.9)	
IgM deposits				
** **0	11 (17.6)	6 (31.6)	17 (20.7)	**0.04**
** **1	13 (20.6)	1 (5.3)	14 (17.1)	
** **2	24 (38.1)	3 (15.8)	27 (32.9)	
** **3	15 (23.8)	9 (47.4)	24 (29.3)	
IgA deposits				
** **0	34 (54.0)	10 (52.6)	44 (53.7)	0.21
** **1	18 (28.6)	2 (10.5)	20 (24.4)	
** **2	7 (11.1)	4 (21.1)	11 (13.4)	
** **3	4 (6.4)	3 (15.8)	7 (8.5)	
C3 deposits				
** **0	5 (7.8)	2 (10.5)	7 (8.4)	0.61
** **1	10 (15.6)	4 (21.1)	14 (16.9)	
** **2	20 (31.3)	3 (15.8)	23 (27.7)	
Complications (%)				
** **Infection	20 (30.8)	3 (15.8)	23 (27.3)	0.20
** **Diabetes mellitus	4 (6.15)	0 (0.0)	4 (4.76)	0.57
** **TB	2 (3.1)	0 (0.0)	2 (2.4)	0.22
** **Death	14 (21.5)	1 (5.3)	15 (17.9)	0.17

IVCYC: intravenous cyclophosphamide; MMF: mycophenolate mofetil; IgG: immunoglobulin G, IgM: immunoglobulin M; IgA: immunoglobulin A; C3: complement 3; grading of deposits (0–none, 1–mild, 2–moderate, and 3–severe); IQR: interquartile range; Tb: tuberculosis.

**Table 3:** : Complete, partial remission and relapse in the two groups after therapy.

	IVCYC OR (95% CI)	MMF OR (95% CI)	Total OR (95% CI)	*p* value^*∗*^
CR at 6 months	18.0 (8.6–31.4)	25.0 (7.3–52.4)	19.7 (10.9–31.3)	0.54
PR at 6 months	58.0 (43.2–71.8)	62.5 (35.4–84.8)	59.1 (46.3–71.0)	0.75
CR + PR	76.0 (61.8–86.9)	87.5 (61.6–98.4)	78.8 (67.0–87.9)	0.33
Relapse at 12 months	8.1 (1.7–21.9)	10.3 (4.3–48.1)	11.5 (4.3–23.4)	0.22

^*∗*^ *p* value for comparison between IVCYC and MMF; CR = complete remission; PR = partial remission; OR: odds ratio; IVCYC: intravenous cyclophosphamide; MMF: mycophenolate mofetil.

**Table 4:** Multivariable analysis for the predictors of remission and mortality.

	Remission		Mortality	
	Odds ratio (95% CI)	*p* value	Odds ratio (95% CI)	*p* value
Age (years)	1.0 (0.9–1.1)	0.43	1.02 (0.94–1.09)	0.69
Gender				
** **Female	1	0.48	1	0.66
** **Male	2.4 (0.2–28.3)		0.60 (0.06–5.87)	
Ethnicity				
** **Black Africans	1	0.62	1	0.59
** **Mixed ancestry	1.5 (0.3–6.5)		0.66 (0.14–3.01)	
Induction regimen				
** **IVCYC	1	0.20	1	0.11
** **MMF	3.2 (0.5–18.0)		0.07 (0.003–1.87)	
Baseline eGFR	1.0 (0.97–1.0)	0.43	0.95 (0.92–0.98)	**0.001**
Baseline uPCR	1.0 (0.99–1.0)	0.26	0.99 (0.99–1.00)	0.15
Interstitial fibrosis	1.7 (0.4–6.7)	0.46	0.34 (0.09–1.24)	0.10
% crescents	3.0 (0.1–68.2)	0.49	0.83 (0.08–8.37)	0.87
% sclerosed glomeruli	0.5 (0.0–14.6)	0.69	0.99 (0.07–12.99)	0.99

IVCYC: intravenous cyclophosphamide, MMF: mycophenolate mofetil, eGFR: estimated glomerular rate; uPCR: urine protein-creatinine ratio.

## Data Availability

The data can be obtained upon request.

## Supplementary Materials

Supplementary Materials Table S1: sensitivity analysis showing multivariable models for the predictors of remission and mortality.
