# Supplementary material for: Comparing the Efficacy and Safety of Induction Therapies for the Treatment of Patients with Proliferative Lupus Nephritis in South Africa
Source: Int J Nephrol. 2020 Oct 19;2020:2412396. doi: 10.1155/2020/2412396 (PMC7591955; doi:10.1155/2020/2412396)
Supplement: Supplementary Materials — Table S1: sensitivity analysis showing multivariable models for the predictors of remission and mortality. [file 2412396.f1.docx]

**Supplementary materials:**

**Supplementary Table legend:**

**Table S1:** Sensitivity analysis showing multivariable models for the predictors of remission and mortality

**Table S1:** Sensitivity analysis showing multivariable models for the predictors of remission and mortality

|  | **Remission** | | **Mortality** | |
| --- | --- | --- | --- | --- |
|  | **Hazard ratio (95% CI)** | **p-value** | **Hazard ratio (95% CI)** | **p-value** |
| Age (years) | 1.10 (0.99-1.23) | 0.06 | 1.03 (0.89-1.19) | 0.71 |
| Ethnicity |  |  |  |  |
| - Black Africans | 1 |  | 1 |  |
| - Mixed ancestry | 0.33 (0.07 – 1.49) | 0.15 | 0.16 (0.03 – 3.41) | 0.36 |
| Induction regimen: |  |  |  |  |
| - IVCYC | 1 |  | 1 |  |
| - MMF | 0.30 (0.06-1.56) | 0.15 | 0.16 (0.01 – 1.98) | 0.15 |
| Baseline eGFR | 0.98 (0.97-1.00) | 0.12 | 0.99 (0.97-1.01) | 0.47 |
| Baseline uPCR | 1.06 (0.10-11.18) | 0.96 | 0.15 (0.003-7.64) | 0.34 |
| Interstitial fibrosis | 0.56 (0.18-1.79) | 0.33 | 0.42 (0.08-2.09) | 0.29 |
| % Crescents | 1.05 (0.87-1.26) | 0.60 | 1.01 (0.78-1.31) | 0.95 |
| % Sclerosed glomeruli | 0.81 (0.43-1.53) | 0.52 | 0.93 (0.54-1.60) | 0.80 |

IVCYC – Intravenous cyclophosphamide, MMF – Mycophenolate mofetil, eGFR= estimated glomerular rate; uPCR=urine protein creatinine ratio
